# Genipin Ameliorates Carbon Tetrachloride-Induced Liver Injury in Mice via the Concomitant Inhibition of Inflammation and Induction of Autophagy

**DOI:** 10.1155/2019/3729051

**Published:** 2019-12-11

**Authors:** Ya Wang, Tianming Zhao, You Deng, Lijun Hou, Xiaofei Fan, Lin Lin, Wei Zhao, Kui Jiang, Chao Sun

**Affiliations:** ^1^Department of Gastroenterology and Hepatology, Tianjin Medical University General Hospital, Anshan Road 154, Heping District, Tianjin 300052, China; ^2^Department of Gastroenterology, Shanxi Academy of Medical Sciences Shanxi Bethune Hospital, Taiyuan 030032, China; ^3^Tianjin Institute of Digestive Disease, Tianjin Medical University General Hospital, Anshan Road 154, Heping District, Tianjin 300052, China; ^4^Department of Gastroenterology, Tianjin Medical University General Hospital Airport Hospital, East Street 6, Tianjin Airport Economic Area, Tianjin 300308, China

## Abstract

Genipin, as the most effective ingredient of various traditional medications, encompasses antioxidative, anti-inflammatory, and antibacterial capacities. More recently, it is suggested that genipin protects against septic liver damage by restoring autophagy. The purpose of the current study was to explore the protective effect of genipin against carbon tetrachloride- (CCl_4_-) induced acute liver injury (ALI) and its underlying molecular machinery. Our results indicated that treatment with genipin significantly reduced CCl_4_-induced hepatotoxicity by ameliorating histological liver changes, decreasing the aspartate aminotransferase and alanine transaminase levels, alleviating the secretion of inflammatory cytokines, and promoting autophagic flux. Moreover, genipin effectively induced the conversion of LC3 and inhibition of p62 accumulation. The liver expressions of ATG5, ATG7, and ATG12 were significantly increased by genipin pretreatment in the ALI mice model. This protective effect may be mediated by the inhibition of mTOR and the activation of p38 MAPK signaling pathways. Meanwhile, genipin attenuated CCl_4_-induced inflammatory response by inhibiting the NF-*κ*B and STAT3 signaling pathway. In addition, pretreatment with autophagy inhibitor 3-methyladenine (3-MA) or inhibition of p38 MAPK by SB203580 abolished the hepatoprotective effect of genipin. Taken together, our study implicates that genipin has a protective potential against CCl_4_-induced hepatotoxicity, which might be strongly associated with the induction of autophagy and the attenuation of inflammatory response.

## 1. Introduction

Acute liver injury (ALI) is a functional liver abnormality that results from various reasons, including viral infection and abuse of drugs or alcohol, as well as ingestion of toxic substances [1, 2]. Extensive or consistent liver injury may result in liver failure or liver cirrhosis. The nature of liver damage has been broadly investigated, but the mechanisms of ALI are still far from being classified [3].

Autophagy initiates with the sequestration of regions of cytosol in double-membrane compartments followed by the formation of autophagosomes and lysosome-based degradation of the contents [4]. It is believed that autophagy serves as an adaptive strategy by which cells can digest damaged organelles and enhance survival by providing energy under bioenergetics-induced stress. Autophagy also represents multiple roles in the regulation of cell death, differentiation, and antimicrobial activities in mammals [5, 6]. In addition, a complicated reciprocal relationship has been observed between autophagy pathway/proteins and inflammation [7]. A recent study indicated that genipin provided protection against flagellin-induced lung inflammation by inhibiting inflammasome-associated cytokine production and inhibiting autophagy [8].

The Chinese herb *Gardeniae fructus* (GF) is an evergreen Rubiaceae shrub, which is widely used in Asian countries as a complementary and alternative therapy [9]. GF extracts have been used for treating inflammation, jaundice, and hepatic disorders in traditional Chinese medicine (TCM) [10]. A variety of TCM preparations contains GF extracts, such as Yin-Chen-Hao-Tang (YCHT) [11], Yin-Zhi-Huang [12], or Huang-Lian-Jie-Du-Tang [13]. Intriguingly, Uji et al. found that the serum concentration of genipin, which has been considered a major active ingredient contributing to the pharmacological effect of YCHT, increased after YCHT administration and showed a positive correlation with the choleretic effect of YCHT [14]. Furthermore, genipin protected against sepsis-induced liver injury by restoring autophagy [15]. However, there is limited information on the core molecular machinery of genipin-induced autophagy and its regulatory signaling in carbon tetrachloride- (CCl_4_-) induced acute liver damage. Collectively, this study is aimed at investigating the hepatoprotective effect of genipin and discovering the underlying mechanisms.

## 2. Materials and Methods

### 2.1. Chemicals and Reagents

CCl_4_ was purchased from Fuyu Chemical Industry Co., Ltd. (Tianjin, China). Genipin and 3-MA was purchased from Sigma-Aldrich (St. Louis, MO, USA).

### 2.2. Animals

C57BL/6 mice (male, 6-8 weeks, 20-22 g) were purchased from the National Institutes for Food and Drug Control (Beijing, China). Mice were housed in a room maintained at a temperature of 23 ± 2°C and relative humidity of 50 ± 10% with a 12 h light-dark cycle. Mice were acclimatized for 1 week prior to use and had free access to food and water during the entire experiments. All animal experiments were approved by the Institutional Animal Care and Use Committee at the Tianjin Medical University General Hospital.

The mice received an intraperitoneal (*i.p*.) injection of a mixture of CCl_4_ (50%) and oil (50%) at a dose of 2 ml·kg^−1^ body weight. The control group was given an intraperitoneal injection of the same value of oil as the CCl_4_ group. The mice were sacrificed at 12, 24, and 48 h after the CCl_4_ injection. The mice received an intravenous injection of genipin or saline (vehicle) via the tail vein 2 h before CCl_4_ exposure. We selected 2.5 mg·kg^−1^ genipin as an optimally effective dose for entire experiments on the basis of previous studies [15]. 3-MA was dissolved in saline (1 mg·kg^−1^) and injected through the tail vein 1 h before genipin treatment to determine the suppression of autophagy with regard to the protective effects of genipin on CCl_4_-induced ALI. Twenty-four mice were randomly divided into four groups as follows (*n* = 6 each group): (1) vehicle-treated normal control (control); (2) vehicle-treated CCl_4_ exposure (CCl_4_); (3) 2.5 mg·kg^−1^ genipin-treated CCl_4_ exposure (CCl_4_+genipin); and (4) 3-MA and genipin-treated CCl_4_ exposure (CCl_4_+genipin+3-MA).

### 2.3. Alanine Transaminase (ALT) and Aspartate Transaminase (AST) Assays

The levels of serum ALT and AST were determined by using an Automated Chemical Analyzer (Hitachi 7080, Hitachi High-Technologies America, Inc.) with the standard diagnostic kits (Shanghai Kehua Bio-Engineering Co., Ltd., Shanghai, China).

### 2.4. Immunohistochemistry and Histological Evaluation

Liver tissue was collected 12, 24, and 48 h after CCl_4_ treatment. A portion of liver tissue was fixed in 10% neutral buffered formalin for histology and immunohistochemistry, and the rest of the sample was used for western blot analysis. Formalin-fixed, paraffin-embedded liver tissues were cut into 5 *μ*m thick sections and stained with hematoxylin and eosin (H&E). The Knodell score was used to grade the severity of the necroinflammatory process [16].

### 2.5. Transmission Electron Microscopy (TEM)

Liver tissues were fixed in 2.5% glutaraldehyde and 4% paraformaldehyde in 100 mM sodium phosphate (pH 7.2). Samples were washed with 100 mM Na cacodylate (pH 7.4), postfixed in 2% osmium tetroxide, and then washed again. The samples were dehydrated in a graded series of ethanol and propylene oxide and embedded in epoxy resin (TAAB 812 Resin; Marivac Industries, Montreal, QC, Canada). Ultrathin (60-70 nm) sections were counterstained with uranyl acetate and lead citrate and viewed using a Hitachi 7600 TEM (Hitachi High-Technologies America, Inc., Schaumburg, IL, USA) equipped with a MacroFire monochrome progressive scan CCD camera (Optronics, Inc., Muskogee, OK, USA) and AMTV image capture software (Advanced Microscopy Techniques, Corp., Danvers, MA, USA).

### 2.6. Cytokine Measurement

Circulating cytokine profiles comprised mice from all four treatment groups. For the cytokine assays, whole blood samples were collected into disposable vacuum blood collection tubes (BD, USA). After 0.5 h of standing in room temperature and being centrifuged at 2000 rpm for 10 min, serum was then obtained. The supernatant was pipetted into EP tubes and stored at -80°C until use. We quantitatively detected the expression level of six circulating cytokines, including IL-1*β*, IL-6, CCL20, IL-10, IL-17A, and IL-33 using MILLIPLEX® MAP Mouse High-Sensitivity Cytokine Panels for a 96-well assay (Millipore Corporation, Billerica MA, USA) on a Luminex platform [17]. Only measurements with CV ≤ 20% were included in the analysis. All cytokine concentrations were analyzed in the same bead suspension to minimize interexperimental variability. For quality assurance, each sample was run twice, and the mean derivation was used as the index value.

### 2.7. Western Blot Analysis

After the designated treatments were implemented, liver tissues and cell pellets were lysed with RIPA buffer supplemented with protease inhibitors. The protein concentration was measured using the BCA protein assay kit. Total proteins (30 *μ*g) were separated via 8-12% SDS-polyacrylamide gel electrophoresis (PAGE) and transferred to nitrocellulose (NC) membranes. The following primary antibodies were employed: primary rabbit antibodies against microtubule-associated protein 1 light chain 3 A/B (LC3A/B) (1 : 1000, no. 12741), p62 (1 : 1000, no. 5114), Atg5 (1 : 1000, no. 12994), Atg7 (1 : 1000, no. 2631), Atg12 (1 : 1000, no. 4180), Beclin-1 (1 : 1000, no. 3738), mTOR (1 : 1000, no. 2983), phospho- (p-) mTOR (1 : 1000, no. 5536), p38 (1 : 1000, No. 8690), p-p38 (1 : 1000, no. 4511), ERK1/2 (1 : 5000, no. 4696), p-ERK1/2 (1 : 2000, no. 4370), Stat3 (1 : 2000, no. 4904), p-Stat3 (1 : 1000, no. 94994), NF-*κ*B p65 (1 : 1000, no. 3036), *β*-actin (1 : 1000, no. 4970) (Cell Signaling Technology, Beverly, MA, USA), JNK (1 : 1000, ab208035), and p-JNK (1 : 5000, ab76572) (Abcam, Cambridge, MA, USA). Peroxidase-conjugated goat anti-rabbit or anti-mouse IgG (1 : 5000) (Zhongshan Golden Bridge Biotechnology, Beijing, China) was employed as the secondary antibodies. The specific protein bands were visualized using the enhanced western luminescent detection kit (Vigorous Biotechnology, Beijing, China). The results were quantified by densitometry using ImageJ software, and the densitometry results were normalized relative to the *β*-actin bands.

### 2.8. Statistical Analysis

All results are presented as means ± standard error of the mean (SEM). The overall significance of the data was examined by two-way analysis of variance. Differences between groups were considered statistically significant at *p* < 0.05 with the appropriate Bonferroni correction made for multiple comparisons.

## 3. Results

### 3.1. Genipin Pretreatment Attenuates CCl_4_-Induced Acute Liver Injury in Mice

**Figure 1:**
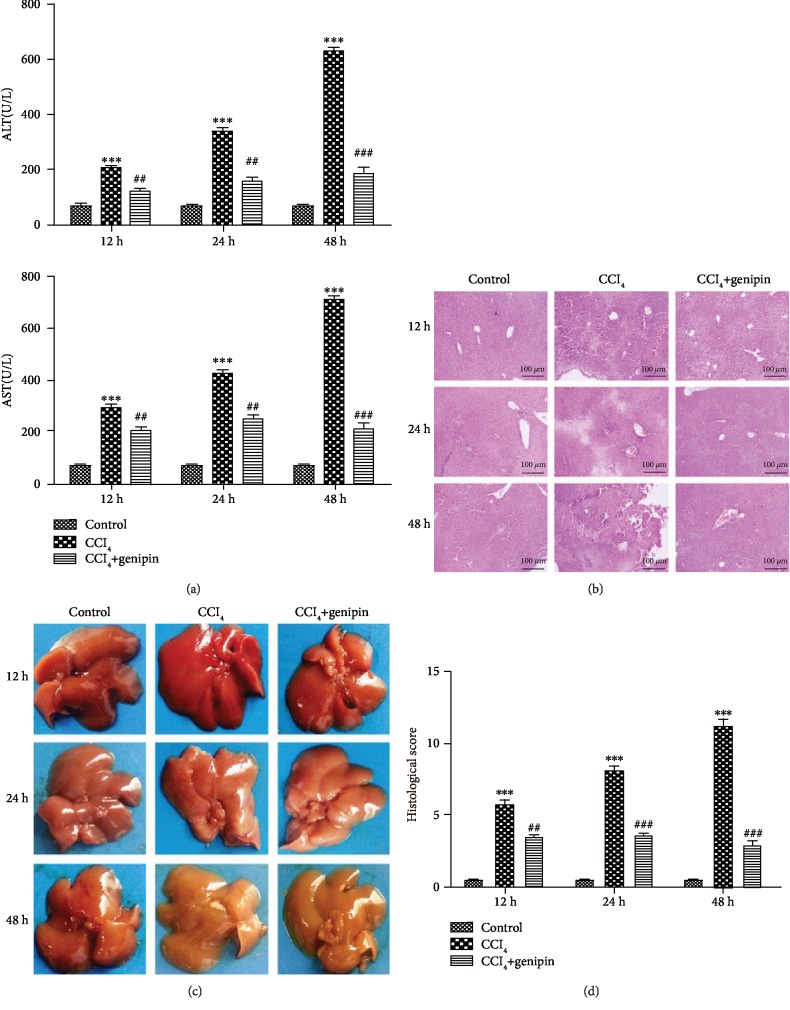
Effects of genipin on serum ALT/AST activity (a), H&E staining (b), macroscopic examination (c), and histological score (d) at 12, 24, and 48 h after CCl_4_ exposure. Mice were intraperitoneally injected a mixture of CCl_4_ (50%) and oil (50%) at a dose of 2 ml·kg^−1^ body weight. Mice received an intravenous injection of 2.5 mg·kg^−1^ genipin 2 h before CCl_4_ exposure. Results are presented as mean ± SEM for six mice per group. Significantly different (^∗∗∗^*p* < 0.001) from the control group. Significantly different (^##^*p* < 0.01 and ^##^*p* < 0.001) from the CCl_4_ group.

First, we evaluated the time course of the hepatoprotective effect of genipin against CCl_4_-induced ALI using the levels of serum ALT and AST, and liver histology as endpoints. As shown in Figure 1(a), the mice from the CCl_4_+genipin group displayed significantly attenuated serum ALT and AST levels when compared with the CCl_4_ group (all *p* < 0.01 or 0.001).

Histological estimation of the livers of mice from the CCl_4_ group revealed more apparent liver injury at 48 h, seen as a large portion of extensive cellular necrosis accompanied with loss of hepatic architecture and infiltration of inflammatory cells (Figure 1(b)). As shown in Figure 1(c), these findings were also confirmed by macroscopic evaluation. Compared with the control group, the histological scores for the CCl_4_ group at 12, 24, and 48 h were all increased to 5.8 ± 1.0, 8.0 ± 0.9, and 11.2 ± 1.1, respectively. Genipin pretreatment significantly diminished the histological scores at 12, 24, and 48 h to 3.3 ± 0.8, 3.5 ± 0.5, and 2.8 ± 0.8, respectively (Figure 1(d)).

### 3.2. The Time Course Changes of Autophagy Flux during CCl_4_-Induced Liver Injury

To evaluate autophagic flux in the liver, we examined changes of protein expression levels regarding LC3-II and p62, which is a polyubiquitin-binding protein known to be sequestered and degraded during autophagy. The level of LC3-II protein expression significantly increased 1.8-fold and 2.1-fold, respectively, compared with that of the control group after 12 and 24 h of CCl_4_ challenge and declined to the control level after 48 h of CCl_4_ challenge (Supplementary [Supplementary-material supplementary-material-1]). Similarly, the level of p62 protein expression significantly increased 3.1-fold, 6.1-fold, and 4.3-fold, respectively, from that of the control group after 12, 24, and 48 h of CCl_4_ exposure. On the basis of serum ALT/AST activity, histological assessment, and autophagy molecules, we selected 48 h after CCl_4_ exposure as the optimal time for further biochemical and molecular studies.

### 3.3. Genipin Pretreatment Promotes Autophagy and Contributes to Hepatocellular Protection against CCl_4_ Exposure

**Figure 2:**
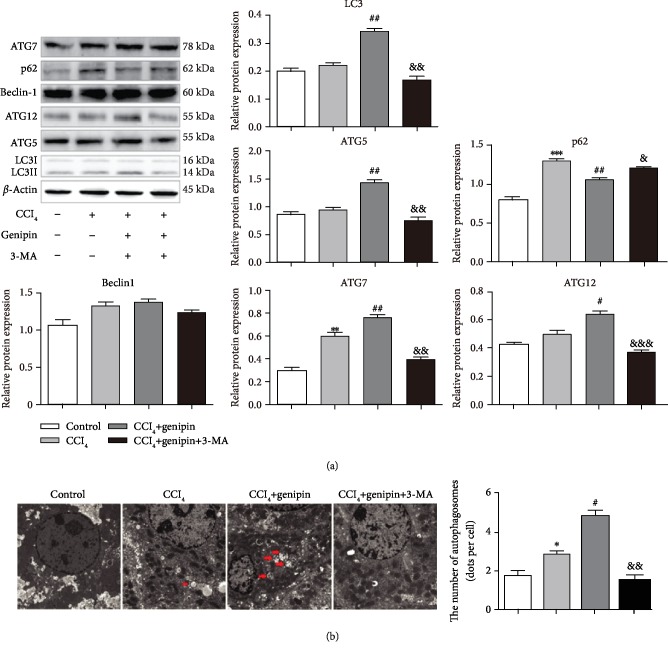
Effect of genipin and 3-MA on LC3-II, p62, ATG5, ATG7, ATG12, and Beclin-1 protein expressions (a) and transmission electron microscopy images (b) at 48 h after CCl_4_ exposure. Mice were intraperitoneally injected a mixture of CCl_4_ (50%) and oil (50%) at a dose of 2 ml·kg^−1^ body weight. Mice received an intravenous injection of 2.5 mg·kg^−1^ genipin 2 h before CCl_4_ exposure. Mice were pretreated with 3-MA before genipin. Results are presented as mean ± SEM for each group. Significantly different (^∗^*p* < 0.05, ^∗∗^*p* < 0.01, and ^∗∗∗^*p* < 0.001) from the control group. Significantly different (^#^*p* < 0.05 and ^##^*p* < 0.01) from the CCl_4_ group. Significantly different (^&^*p* < 0.05, ^&&^*p* < 0.01, and ^&&&^*p* < 0.001) from the CCl_4_+genipin group.

To investigate the role of genipin regarding autophagy activation during CCl_4_ exposure in hepatoprotection, we examined the autophagy-related protein expression of liver tissue by western blot analysis and utilized autophagy inhibitor 3-MA. First of all, we revealed no impact of 3-MA in the mice model (Supplementary [Supplementary-material supplementary-material-1]). As shown in Figure 2(a), the results showed that ATG7 and p62 were dramatically increased in the CCl_4_ group, while no significant changes were found with respect to protein expression of Beclin-1, LC3-II, ATG5, or ATG12. Moreover, treatment of genipin significantly increased the expression levels of LC3-II, ATG5, ATG7, and ATG12 to 1.4-fold, 1.4-fold, 1.3-fold, and 1.3-fold, respectively, compared with that of the 48 h CCl_4_ exposure group. In contrast, the increased level of p62 protein was attenuated by genipin. Furthermore, treatment with 3-MA abrogated the elevated level of LC3-II, ATG5, ATG7, and ATG12 and reversed the attenuated level of p62 by genipin. To confirm our western blot analysis, we observed autophagic vacuoles, including autophagosomes and autolysosomes, by TEM (Figure 2(b)). We characterized autophagic vacuoles by double-membrane structures containing cytoplasm or undigested organelles. Compared with the basal level of autophagic vacuoles in the control group, the number of autophagic vacuoles slightly increased after CCl_4_ exposure, which was augmented by genipin. 3-MA also abolished this effect by inhibiting the autophagy process.

**Figure 3:**
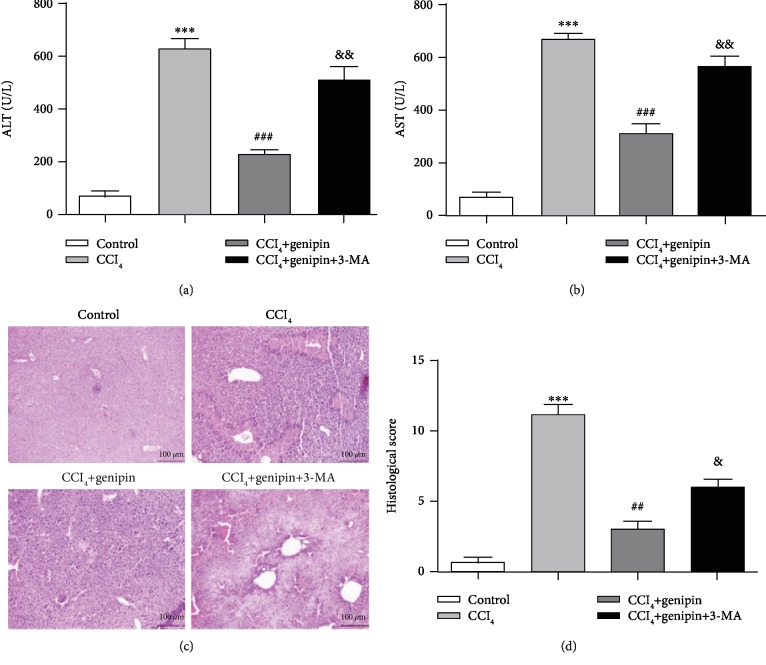
Effect of genipin and 3-MA on serum ALT activity (a), AST activity (b), H&E staining (c), and histological score (d). Mice were intraperitoneally injected a mixture of CCl_4_ (50%) and oil (50%) at a dose of 2 ml·kg^−1^ body weight. Mice received an intravenous injection of 2.5 mg·kg^−1^ genipin 2 h before CCl_4_ exposure. Mice were pretreated with 3-MA before genipin. Significantly different (^∗∗∗^*p* < 0.001) from the control group. Significantly different (^##^*p* < 0.01 and ^###^*p* < 0.001) from the CCl_4_ group. Significantly different (^&^*p* < 0.05 and ^&&^*p* < 0.01) from the CCl_4_+genipin group.

Moreover, 3-MA also reversed the hepatoprotection of genipin against CCl_4_-induced ALI as indicated by increased ALT/AST activity, aggravated histological score, and morphologic observations with H&E staining (Figure 3). Liver section isolated from the CCl_4_+genipin+3-MA group showed multiple and extensive portions of portal inflammation and hepatocellular necrosis, as well as a moderate increase in inflammatory cell infiltration and congestion.

### 3.4. Genipin Enhances Autophagy via mTOR Inhibition and p38 MAPK Activation during CCl_4_-Induced Liver Injury

**Figure 4:**
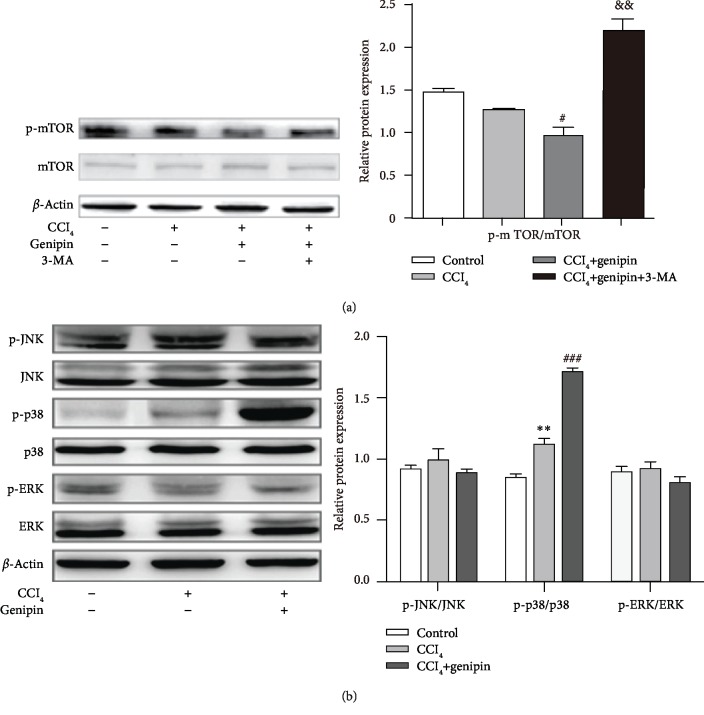
Effect of genipin and/or 3-MA on mTOR (a) and p-JNK, p-p38, and p-ERK (b) protein expressions at 48 h after CCl_4_ exposure. Mice were intraperitoneally injected a mixture of CCl_4_ (50%) and oil (50%) at a dose of 2 ml·kg^−1^ body weight. Mice received an intravenous injection of 2.5 mg·kg^−1^ genipin 2 h before CCl_4_ exposure. Mice were pretreated with 3-MA before genipin. Significantly different (^∗∗^*p* < 0.01) from the control group. Significantly different (^#^*p* < 0.05 and ^###^*p* < 0.001) from the CCl_4_ group. Significantly different (^&&^*p* < 0.01) from the CCl_4_+genipin group.

**Figure 5:**
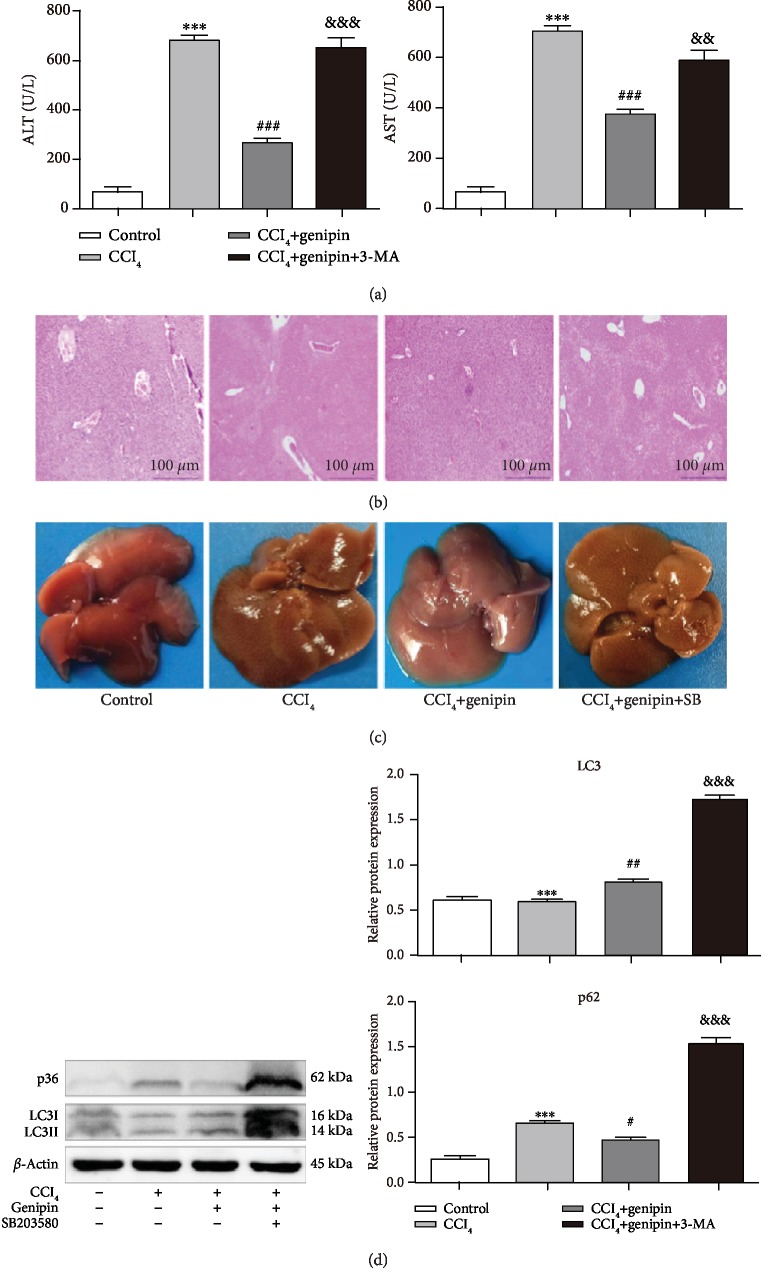
Effect of genipin and SB203580 on serum ALT/AST activity (a), H&E staining (b), macroscopic examination (c), and LC3-II and p62 protein expressions (d). Mice were intraperitoneally injected a mixture of CCl_4_ (50%) and oil (50%) at a dose of 2 ml·kg^−1^ body weight. Mice received an intravenous injection of 2.5 mg·kg^−1^ genipin 2 h before CCl_4_ exposure. Mice were pretreated with SB203580 before genipin. Significantly different (^∗∗∗^*p* < 0.001) from the control group. Significantly different (^#^*p* < 0.05, ^##^*p* < 0.01, and ^###^*p* < 0.001) from the CCl_4_ group. Significantly different (^&&^*p* < 0.01 and ^&&&^*p* < 0.001) from the CCl_4_+genipin group.

To elucidate the molecular mechanisms by which genipin affects autophagy in CCl_4_-induced liver injury, we investigated the involvement of the mTOR and MAPK pathway [18]. As shown in Figure 4, in the CCl_4_+genipin group, the expression level of p-mTOR/mTOR decreased to approximately 80% and 70% that of the control and CCl_4_ exposure group, respectively, and these decreases were attenuated by 3-MA. Moreover, the level of p-p38 MAPK protein expression increased 1.3-fold at 48 h CCl_4_ challenge, and genipin further increased the level of p-p38. There were no differences in the levels of p-JNK or p-ERK among any of the experimental groups. To confirm genipin-induced autophagy via p38 MAPK activation, SB203580 abolished the protective effect of genipin as indicated by increased ALT/AST activity and aggregated histological score (Figure 5). Accordingly, SB203580 augmented genipin-induced LC3-II accumulation and dramatically reversed genipin-induced p62 degradation.

### 3.5. Genipin Pretreatment Affects CCl_4_-Induced Inflammatory Responses

**Figure 6:**
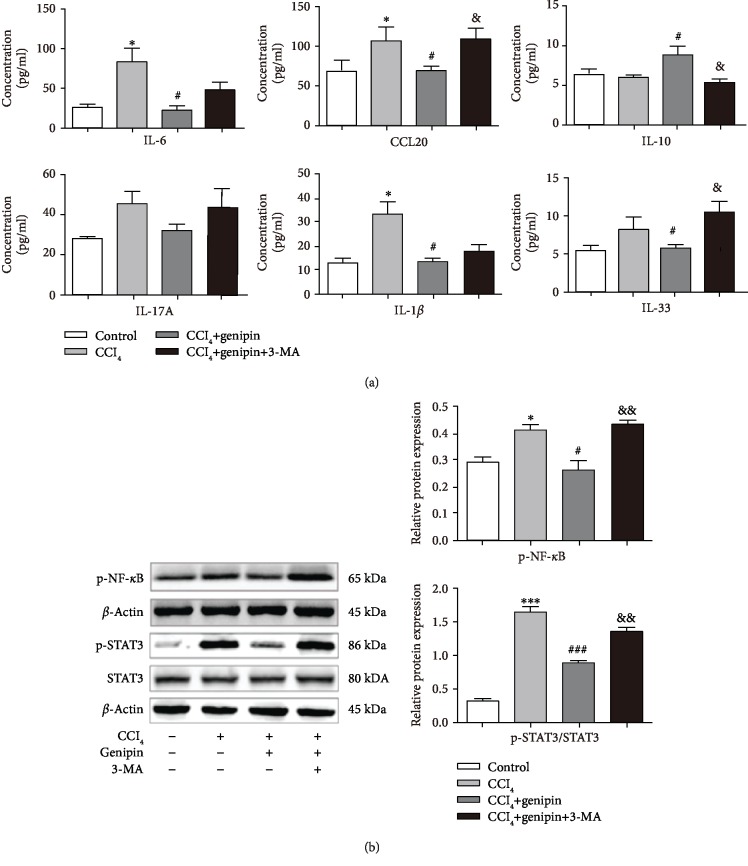
Effect of genipin and/or 3-MA on serum inflammatory parameters (a) and NF-*κ*B and p-STAT3 (b) protein expressions at 48 h after CCl_4_ exposure. Mice were intraperitoneally injected a mixture of CCl_4_ (50%) and oil (50%) at a dose of 2 ml·kg^−1^ body weight. Mice received an intravenous injection of 2.5 mg·kg^−1^ genipin 2 h before CCl_4_ exposure. Mice were pretreated with 3-MA before genipin. Significantly different (^∗^*p* < 0.05 and ^∗∗∗^*p* < 0.001) from the control group. Significantly different (^#^*p* < 0.05 and ^###^*p* < 0.001) from the CCl_4_ group. Significantly different (^&^*p* < 0.05 and ^&&&^*p* < 0.001) from the CCl_4_+genipin group.

We measured the serum levels of several cytokines by using Milliplex, in order to investigate the impact of genipin pretreatment on CCl_4_-induced liver inflammatory responses. In comparison with mice from the control group, mice from the CCl_4_ group showed significantly increased serum levels of IL-6, IL-1*β*, and CCL20. The serum levels of IL-17A and IL-33 were also elevated due to CCl_4_ exposure but without statistical significance. Genipin pretreatment markedly decreased the levels of IL-6, IL-1*β*, and CCL20. Furthermore, 3-MA abrogated the anti-inflammatory effects of genipin against CCl_4_-induced hepatic inflammation responses as indicated by increased IL-6, CCL20, IL-17A, and IL-33 levels compared with genipin-pretreated CCl_4_-exposed animals (Figure 6). Intriguingly, we found that the expression level of IL-10, as a robust anti-inflammation indicator, was not influenced by CCl_4_ exposure. Genipin pretreatment dramatically increased the levels of IL-10, and this effect was reversed by 3-MA.

NF-*κ*B, a transcriptional factor, is implicated in the regulation of several genes coding for mediators of inflammatory responses. Once activated, it is dissociated from I-*κ*B and is translocated into the nucleus, where it initiated the transcriptional upregulation of many inflammatory mediators. In line with this, at 48 h of CCl_4_ exposure, the nuclear protein level of phosphorylated NF-*κ*B (p-NF-*κ*B) was increased about 1.2-fold over the control level. The elevated level of phosphor-NF-*κ*B was dramatically reduced to the control level by genipin treatment. In contrast, this effect was abolished by 3-MA pretreatment.

STAT3 is a cytoplasmic signal transcription factor belonging to the signal transducer and activators of transcription family (STATs). STAT3 activation was reported to play a pivotal role in CCl_4_-induced hepatotoxicity in rodents. In the current study, the active form p-STAT3/total STAT3 increased about 5.1-fold in the CCl_4_ treatment over the control group. A sharp decrease of p-STAT3 was observed by the administration of genipin (approximate 50%), and this effect was abrogated by 3-MA pretreatment.

## 4. Discussion

Autophagy plays important roles in cell survival as well as in the regulation of cell death, which is essential for the maintenance of liver functions [19, 20]. Growing evidence indicates that the modulation of autophagy affects the progression of liver injury. More recently, genipin, as a major active ingredient of *Gardeniae Fructus*, has been proven to have a dual-effect of hepatic autophagy. Yu et al. showed that genipin-inhibited autophagy leading to NLRP3-dependent IL-1*β* production and neutrophil flux against LPS induced murine peritonitis [8]. By contrast, another report indicates that genipin restores the impaired autophagic flux for the prevention of sepsis-induced liver damage [15]. Thus, controversy remains about the effect of genipin on autophagy and the molecular mechanisms are still elusive. Accordingly, we investigated the exact role of autophagy and its possible mechanism of regulation by genipin in mouse liver injury induced by CCl_4_ exposure.

Previous reports indicated that autophagic flux is blocked in response to CCl_4_ treatment, as indicated by an increase in LC3-II and p62 protein [21, 22]. Accordingly, our study showed that LC3-II protein expression increased 12 h after CCl_4_ exposure and declined by 48 h and the p62 protein expression peaked 24 h after CCl_4_ exposure and declined by 48 h after CCl_4_ challenge. LC3-II is only present on mature autophagosomes, and p62, a substrate protein, can recognize the ubiquitinated protein aggregates and directly bind to the LC3-II-specific motif. The accumulation of p62 is indicative of impaired autophagic flux, since p62 is degraded with the autophagic cargo in the autolysosome. The histological and biochemical examination revealed most evident liver damage at 48 h on account of elevated serum ALT/AST activity as well as histological score. Collectively, these findings suggested that autophagy may be induced during consistent liver injury but that the fusion of autophagosomes with lysosomes may be blocked by CCl_4_ treatment. In this study, genipin pretreatment augmented the level of LC3-II protein and dramatically decreased the level of p62 protein. In order to investigate the exact role of autophagy in liver injury, we implemented 3-MA, a class-III PI3K inhibitor. Treatment with 3-MA decreased LC3-II protein expression and enhanced p62 protein expression and reversed the hepatoprotection conferred by genipin, as indicated by a remarkable increase in serum ALT/AST as well as aggravated histological presence. These results were confirmed by TEM images representing that genipin increased the number of autophagic vacuoles.

Beclin-1, ATG5, ATG7, and ATG12 take part in the initiation, extension, and closure of an autophagic vesicle, respectively, in the formation of autophagosomes [23]. Specially, ATG5-ATG12 conjugates are localized to the preautophagosomal structure and the convex surface of the isolation membrane, and ATG7 is a key factor in the ubiquitin-like pathway of LC3 lipidation. In this study, genipin could upregulate ATG5, ATG7, and ATG12, implicating genipin as responsible for enhancing the phagophore elongation and autophagosome maturation. Similar to a previous study, the level of Beclin-1 was not affected among experimental groups. One possible explanation for this observation is that Beclin-1 likely interacts with various activator/inhibitor proteins, including Vps34, HMGB1, and Rubicon, which modulate the autophagy process [24]. The precise role of the Beclin-1 complex in response to CCl_4_ exposure should be elucidated further.

The modulation of autophagy by genipin in liver damage is a novel finding, yet the need to identify the signaling pathway through which genipin triggers autophagy remains. Intriguingly, Cho et al. showed that genipin protected against sepsis-induced liver injury through the downregulation of calpain but not mTOR, by enhancing autophagy machinery [15]. However, accumulating evidence implicates that autophagy can be regulated by MAPK and mTOR signaling [25–27]. Shin et al. showed that activated autophagy by nitric oxide contributes to the hepatoprotective effects through p38 and ERK activation in an animal model of ischemia/reperfusion injury [28]. Moreover, overexpression of p38 MAPK rendered colorectal cancer cell survival against the cytotoxicity of drugs by enhancing autophagy [29]. In the current study, p38 MAPK were markedly stimulated by genipin, while the levels of ERK and JNK were not affected. To clarify whether genipin-induced autophagy is associated with the activation of p38 MAPK, we pretreated mice with SB203580 in CCl_4_-induced liver injury. We found that pretreatment with SB203580 abolished genipin-induced autophagy, as evidenced by the remarkable accumulation of LC3-II and p62 protein expressions. Collectively, our results implicate that genipin-enhanced autophagy might be associated with the inactivation of mTOR and activation of p38 MAPK signaling.

Enhanced autophagy contributes to the inhibition of inflammation, including the downregulation of the interferon response and the suppression of inflammasome-dependent cytokines [30, 31]. Additionally, autophagy also interferes with immune cell selection [32]. Collectively, autophagy can influence both inflammation and immune system findings. The inflammatory response plays a major role in CCl_4_-induced hepatotoxicity, and CCl_4_ metabolic activation results in excessive proinflammatory cytokines (such as IL-1*β* and IL-6), leading to inflammatory formation [33]. In the present study, we found that genipin suppressed hepatic inflammatory response but the inhibition of autophagy reversed the anti-inflammation effect of genipin. Genipin pretreatment not only evidently reduced the serum levels of IL-6, IL-1*β*, and CCL20 but also enhanced the level of IL-10. To further explore the mechanism of the inhibitory effect of genipin on liver inflammation response, we detected the hepatic expression of NF-*κ*B and STAT3, major proteins regulating proinflammatory genes [34]. The results indicated that genipin significantly downregulated the hepatic expression of NF-*κ*B p65 in the nucleus and STAT3 in cytoplasm. Intriguingly, autophagy inhibitor 3-MA reversed this alteration as well as serum proinflammatory cytokines.

Autophagy has now been identified as a main regulator of inflammasomes. Jounai et al. implicated that several inflammasomes, such as NLRC4, NLRP3, NLRP4, and NLRP10 could interact with Beclin-1, while NLRP4 had a strong affinity to the Beclin-1 evolutionally conserved domain [35]. NLRP4 transiently dissociated from Beclin-1 and negatively regulate the autophagic process. On the other hand, autophagy itself can promote inflammasome activities. For instance, Wang et al. found that AIM2 inflammasomes could colocalize with microtubule organizing centers and autophagosomes, and EB-1-mediated AIM2 inflammasome complex activation led to autophagy and IL-1*β* secretion in an LC3-dependent fashion [36]. Intriguingly, a recent study revealed genipin-inhibited autophagy, leading to the inhibition of the NLRC4/NLRP3 inflammasome and subsequently impaired IL-1*β* production and caspase-1 activation [8]. Furthermore, Rajanbabu et al. found that genipin suppressed NLRP3 inflammasome activation through UCP2 and ATP- or H_2_O_2_-mediated IL-1*β* release [37]. Seo et al. indicated that genipin attenuated GalN/LPS-induced increases in the protein expression levels of NLRP3, ASC, and caspase-1, inflammasome components, and levels of liver and serum IL-1*β* [38]. Thus, further work is therefore warranted to broadly investigate whether and how genipin impacts on the complicated network between autophagy and inflammasomes.

In conclusion, this study demonstrated that genipin exhibited hepatoprotection against CCl_4_-induced ALI. This effect was related to its anti-inflammation and enhancement of autophagy flux, which might be mediated by mTOR and p38 MAPK signaling pathways. Collectively, our findings provide novel mechanistic insight into genipin pretreatment regarding amelioration of liver injury.

## Data Availability

The data used to support the findings of this study are included within the article.

## Abbreviations

CCl_4_: Carbon tetrachloride
ALI: Acute liver injury
3-MA: 3-methyladenine
TCM: Traditional Chinese medicine
YCHT: Yin-Chen-Hao-Tang
ALT: Alanine transaminase
AST: Aspartate transaminase
H&E: Hematoxylin and eosin
TEM: Transmission electron microscopy
SEM: Standard error of the mean.

## Supplementary Materials

Supplementary Materials Graphical abstract: genipin significantly reduced CCl_4_-induced hepatotoxicity by enhancing autophagic flux, which was indicative of increased expression of ATG5, ATG7, and ATG12. Moreover, genipin effectively induced the conversion of LC3 and inhibition of p62 accumulation. This protective effect may be mediated by inhibition of mTOR and activation of p38 MAPK signaling pathways. Meanwhile, genipin attenuated CCl_4_-induced inflammatory response by inhibiting the NF-*κ*B and STAT3 signaling pathway. Supplementary Figure 1: the level of LC3-II protein expression significantly increased 1.8-fold and 2.1-fold, respectively, compared with that of the control group after 12 and 24 h of CCl_4_ challenge and declined to the control level after 48 h of CCl_4_ challenge. Similarly, the level of p62 protein expression significantly increased 3.1-fold, 6.1-fold, and 4.3-fold, respectively, from that of the control group after 12, 24, and 48 h of CCl_4_ exposure. Supplementary Figure 2: the sole administration of 3-MA has no impact on serum ALT/AST (A), macroscopic and histological estimation (B), and protein expression levels of LC3/p62 (C) in the CCl_4_-exposed mice model
