# Supplementary material for: Genipin Ameliorates Carbon Tetrachloride-Induced Liver Injury in Mice via the Concomitant Inhibition of Inflammation and Induction of Autophagy
Source: Oxid Med Cell Longev. 2019 Dec 11;2019:3729051. doi: 10.1155/2019/3729051 (PMC6927019; doi:10.1155/2019/3729051)
Supplement: Supplementary Materials — Graphical abstract: genipin significantly reduced CCl4-induced hepatotoxicity by enhancing autophagic flux, which was indicative of increased expression of ATG5, ATG7, and ATG12. Moreover, genipin effectively induced the conversion of LC3 and inhibition of p62 accumulation. This protective effect may be mediated by inhibition of mTOR and activation of p38 MAPK signaling pathways. Meanwhile, genipin attenuated CCl4-induced inflammatory response by inhibiting the NF-κB and STAT3 signaling pathway. Supplementary Figure 1: the level of LC3-II protein expression significantly increased 1.8-fold and 2.1-fold, respectively, compared with that of the control group after 12 and 24 h of CCl4 challenge and declined to the control level after 48 h of CCl4 challenge. Similarly, the level of p62 protein expression significantly increased 3.1-fold, 6.1-fold, and 4.3-fold, respectively, from that of the control group after 12, 24, and 48 h of CCl4 exposure. Supplementary Figure 2: the sole administration of 3-MA has no impact on serum ALT/AST (A), macroscopic and histological estimation (B), and protein expression levels of LC3/p62 (C) in the CCl4-exposed mice model [file 3729051.f1.docx]

**Graphical abstract**


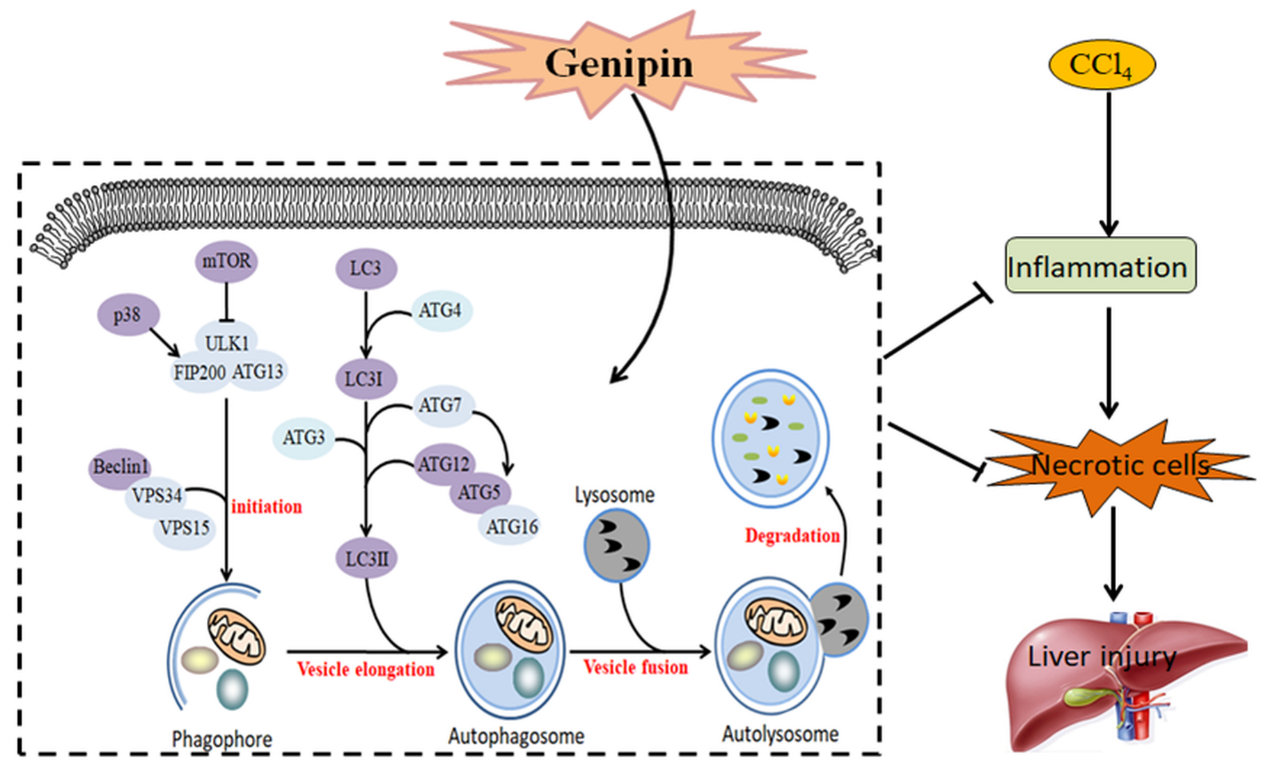


**Supplementary Fig. 1**


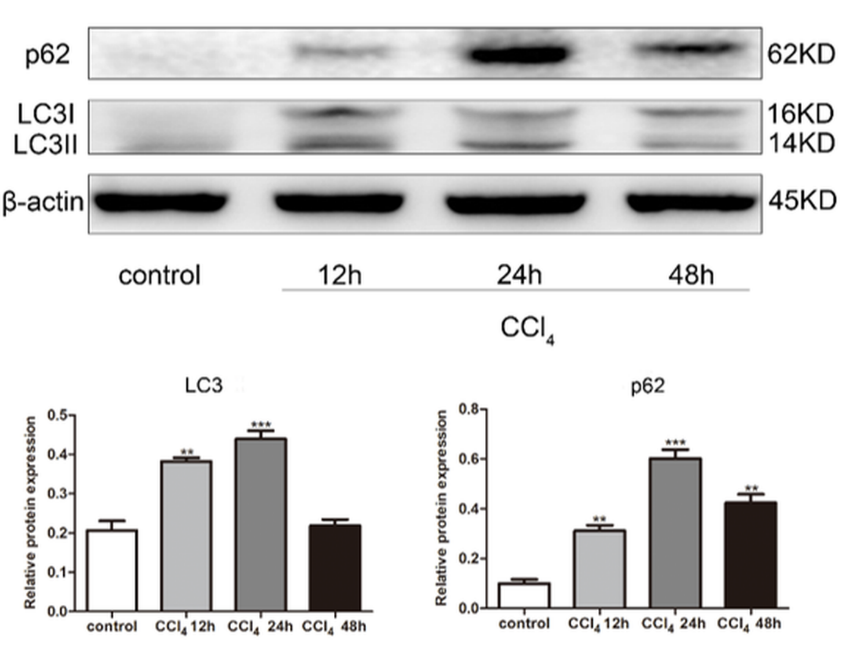


The time course changes of autophagy flux during CCl_4_-induced liver injury

**Supplementary Fig. 2**


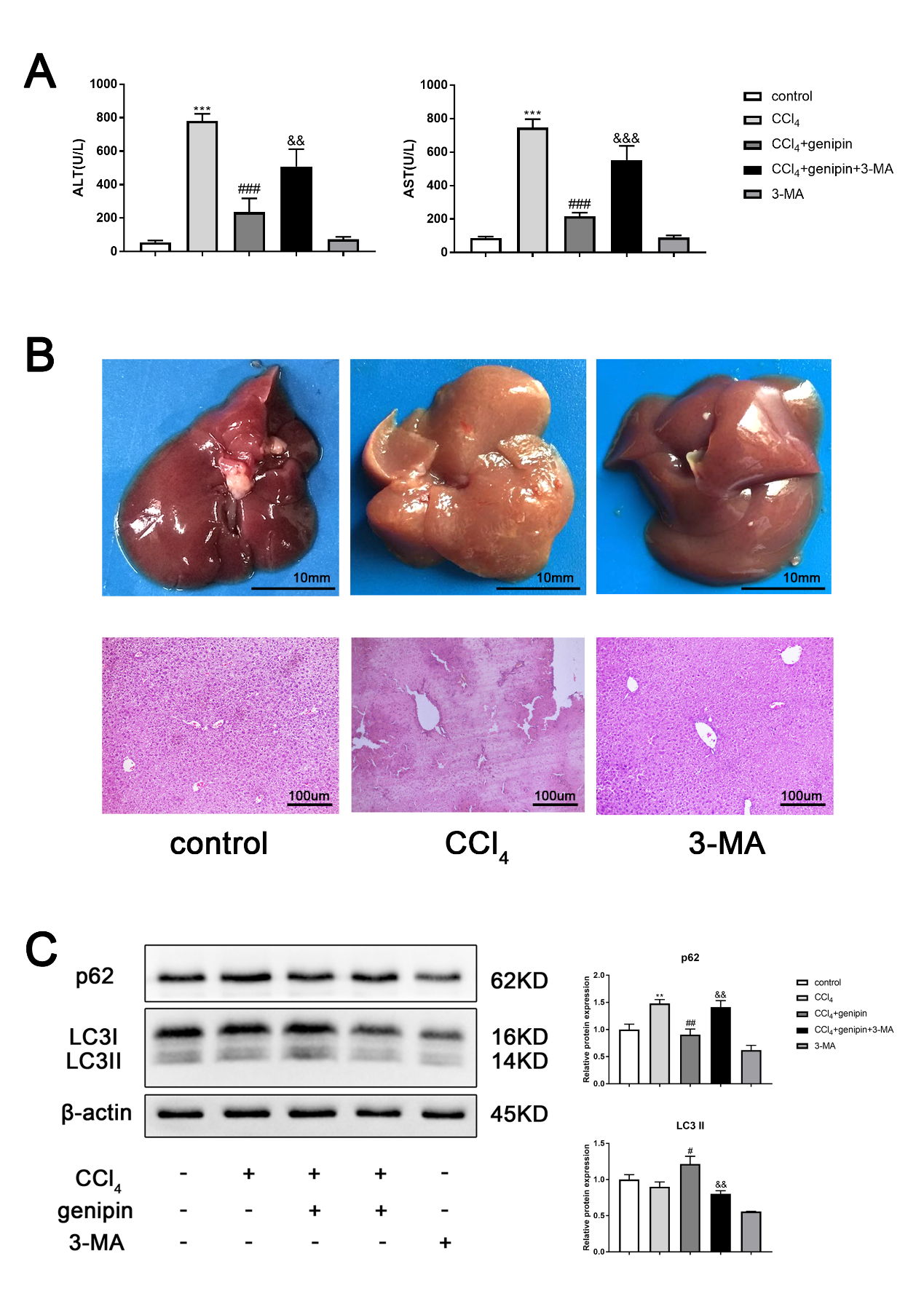


The impact of 3-MA in the mice model
